# HiC4D-SPOT: a spatiotemporal outlier detection tool for Hi-C data

**DOI:** 10.1093/bib/bbaf341

**Published:** 2025-07-16

**Authors:** Bishal Shrestha, Zheng Wang

**Affiliations:** Department of Computer Science, University of Miami, 1365 Memorial Drive, Coral Gables, FL 33146, United States; Department of Computer Science, University of Miami, 1365 Memorial Drive, Coral Gables, FL 33146, United States

**Keywords:** Hi-C, anomaly detection, spatiotemporal autoencoder, ConvLSTM, spatiotemporal dynamics

## Abstract

The 3D organization of chromatin is essential for the functioning of cellular processes, including transcriptional regulation, genome integrity, chromatin accessibility, and higher order nuclear architecture. However, detecting anomalous chromatin interactions in spatiotemporal Hi-C data remains a significant challenge. We present HiC4D-SPOT, an unsupervised deep-learning framework that models chromatin dynamics using a ConvLSTM-based autoencoder to identify structural anomalies. Benchmarking results demonstrate high reconstruction fidelity, with Pearson Correlation Coefficient and Spearman Correlation Coefficient values of 0.9, while accurately detecting deviations linked to temporal inconsistencies, topologically associating domain (TAD) and loop perturbations, and significant chromatin remodeling events. HiC4D-SPOT successfully identifies swapped time points in a time-swap experiment, captures simulated TAD and loop disruptions with high confidence scores and statistical significance of 0.01, and detects HERV-H boundary weakening during cardiomyocyte differentiation, as well as cohesin-mediated loop loss and recovery—aligning with experimentally observed chromatin remodeling events. These findings establish HiC4D-SPOT as an efficient tool for analyzing 3D chromatin dynamics, enabling the detection of biologically significant structural anomalies in spatiotemporal Hi-C data.

## Introduction

DNA, the genetic code, is a long polynucleotide chain intricately folded into chromatin, enabling the cell to condense genomic material into a small volume while preserving essential loci accessibility for vital biological processes [1, 2]. The dynamic and responsive nature of chromatin fold allows 3D organization of the DNA into progressively complex higher order structures that facilitate cell functions at the gene level and global nuclear level, including gene regulation, chromosome morphogenesis, genome stability, and genome transmission [3, 4]. The comprehension of continuous remodeling of 3D chromatin structure provides critical insights into the spatial organization of the genome, elucidating the intricate interplay between the genome and its nuclear environment [5, 6].

Hi-C is a high-throughput, chromosome conformation capture (3C)-based, genome-wide sequencing technology that captures the 3D organization of the genome by detecting all possible pairwise physical interactions among chromatin fragments [7]. Hi-C technique involves the cross-linking of chromatin, digestion of the cross-linked DNA, ligation of the spatially proximal digested fragments, and sequencing of the ligated fragments to identify the interacting chromatin regions [8]. Over the past decade, Hi-C has evolved from a pioneering technique to a powerful benchmark tool for exploring the 3D genome architecture at multiple scales and resolutions [9], revolutionizing the field of genomics by enabling the identification and study of chromatin loops, topologically associating domains (TADs), and compartments, thereby providing a comprehensive spatial context for understanding the 3D structure of the genome [10].

The advent of multi-omics and single-cell sequencing technologies has further elevated the capabilities of Hi-C by enhancing the exploration of complex genomic arrangements and regulations from multiple molecular perspectives: metabolomics, proteomics, transcriptomics, and epigenomics [11–13]. Additionally, by capturing changes in 3D chromatin organization across conditions, cell types, or time points, spatiotemporal Hi-C data illuminate the dynamic processes of genome folding, revealing how chromatin interactions evolve during development, differentiation, and disease states [14–17].

Hi-C data, while offering crucial 3D genomic insights, are inherently high-dimensional, sparse, and noisy, often exacerbated by experiment-induced technical biases introduced during crosslinking, ligation, and mapping [8, 18–21]. Moreover, achieving adequate resolution in Hi-C experiments demands substantial sequencing resources, making large-scale or repeated experiments both expensive and time-consuming, thereby highlighting the importance of the identification of atypical regions in Hi-C data [22].

In order to identify these atypical spatiotemporal dynamics of chromatin structure, it is essential to detect anomalies in the data, which refers to the process of identifying data points, patterns, or observations that deviate significantly from expected distribution [23, 24]. The detection of anomalies in the spatiotemporal Hi-C data can be crucial in identifying potential technical biases, experimental artifacts, or biologically significant deviations such as structural variations (SVs) that may indicate novel insights into chromatin structure and function [25]. While anomaly detection has been extensively studied in various domains, including computer vision, finance, and healthcare, its application in the context of Hi-C data remains unexplored [23, 24, 26–29]. Previous work, such as HiC4D [30], addressed the spatiotemporal dynamics of Hi-C data by forecasting future contact matrices from earlier time points, and thus differs in objective from identifying abnormal structural changes within observed Hi-C sequences. Furthermore, HiC4D employs a residual ConvLSTM architecture trained in a supervised manner to forecast future chromatin contact matrices, diverging from the architectural and functional design of HiC4D-SPOT, which utilizes spatial encoding and decoding layers surrounding the ConvLSTM layers to perform unsupervised detection of deviations in spatiotemporal Hi-C data.

Moreover, despite the availability of an enormous corpus of genomic data, the lack of annotated data presents a significant challenge in the field of genomics and bioinformatics [31–33]. Unsupervised learning techniques play a crucial role in addressing this challenge by enabling the analysis of unlabeled data to uncover hidden patterns, detect anomalies, and gain insights without prior assumptions [34–36].

To address this gap, we developed a novel unsupervised deep-learning method centered on spatiotemporal autoencoder architecture to identify anomalous interaction patterns across temporal Hi-C data. The spatiotemporal autoencoder architecture dynamically models both spatial organization and temporal shifts in Hi-C data, directly detecting anomalies linked to SVs, experimental inconsistencies, and biologically relevant irregularities. Experimental results validate the method’s ability to identify these deviations, offering genomic and bioinformatics researchers a focused analytical resource.

## Materials and methods

### Overview

**Figure 1:**
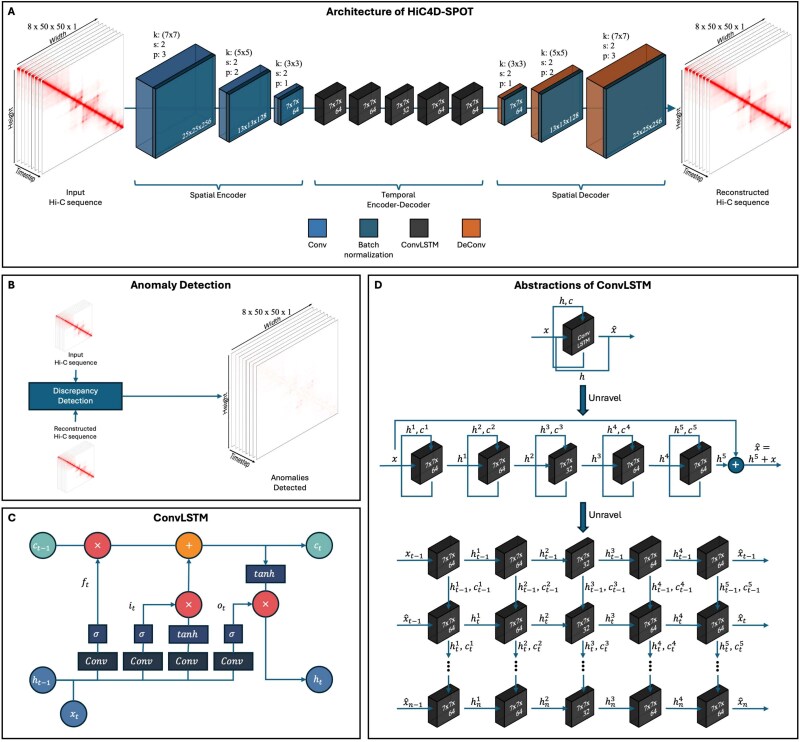
The overall architecture of HiC4D-SPOT for spatiotemporal anomaly detection in spatiotemporal Hi-C data: (A) the pipeline comprises three blocks—a spatial encoder, a ConvLSTM-based temporal encoder–decoder, and a spatial decoder, transforming Hi-C input into its reconstructed output; (B) anomaly detection is performed by computing discrepancies between the input and reconstructed Hi-C sequences to identify anomalous regions; (C) the ConvLSTM module is detailed, showing the internal gating mechanisms and state transitions that capture spatiotemporal dependencies; (D) the abstraction levels of ConvLSTM, unraveling the RNN structure to reveal the information flow between the cells.

The architecture of HiC4D-SPOT in Fig. 1 elucidates the artificial intelligence (AI)-driven spatiotemporal anomaly detection framework for Hi-C data, comprising a spatial encoder, a ConvLSTM-based temporal encoder–decoder, and a spatial decoder. The spatiotemporal Hi-C data are transformed into a compact representation by the spatial encoder, which is then passed to the ConvLSTM-based temporal encoder–decoder to model temporal dependencies across consecutive frames. The spatial decoder reconstructs the original spatial resolution, and anomalies are detected via the reconstruction loss. The abstraction levels of ConvLSTM in Fig. 1 unravel the recurrent neural network (RNN) structure, revealing the information flow between the cells, which is critical for detecting structural anomalies in Hi-C data. Later sections will provide detailed information on the datasets, the spatiotemporal autoencoder architecture, and the implementation.

### Spatiotemporal Hi-C datasets

#### Real Hi-C datasets

In this study, four spatiotemporal Hi-C datasets were used, each with varying numbers of time points, resolutions, and biological contexts. We first utilized a publicly available Hi-C dataset of preimplantation mouse embryos from early mammalian development [37], referred to as the Du dataset, hosted under Gene Expression Omnibus (GEO) accession GSE82185, comprising Hi-C libraries from multiple embryonic stages: gametes (sperm and MII oocyte), pronuclear stage 5 zygotes, early two-cell, late two-cell, eight-cell, inner cell mass, and mouse embryonic stem cells. To form our spatiotemporal series, we selected the last six developmental stages (i.e., excluding gametes). All valid intrachromosomal Hi-C read pairs (>20 kb) were uniformly downsampled to 115 million reads per stage to balance sequencing depth across time points. We used the mm9 reference genome, focusing our analyses on chromosomes 1–19 and X. Each stage’s downsampled dataset was converted into a .cool file at 40 kb resolution using the cooler package (v0.10.0) [38]. From each balanced contact map, 50$\times $50 submatrices were extracted along the diagonal in 2 Mb windows with a stride of 3 bins to capture TAD-scale structural features. To counteract the outliers, the contacts were clipped in the range of 0–100 and then scaled with min-max normalization. For training and evaluation, we split chromosomes into three sets: chromosome 19 for validation, chromosomes 2 and 6 for testing, and all remaining autosomes plus chrX for training.

The second dataset, referred to as the Reed dataset, was derived from a human cell line dynamics study [16], hosted publicly under GEO SuperSeries GSE201376, where eight time points (0, 0.5, 1, 1.5, 2, 4, 6, and 24 h) Hi-C data were available in .hic format, which was then converted to .cool using hic2cool (v1.0.1) [39] at a 10 kb resolution. These contact maps, aligned to the hg19 reference genome, focusing our analysis on 22 autosomes and X, were then balanced via the iterative correction (ICE) method [18, 38]. Analogous to the first dataset, we extracted 50$\times $50 submatrices along each chromosome diagonal with a stride of 25, along with a 25-bin stride to the right until the 2Mb region was covered. To counteract the outliers, the values were clipped in the range of 0–0.02 and then scaled with min-max normalization. For model training and evaluation, we performed a chromosome-based split into three sets: chromosome 19 for validation, chromosomes 2 and 6 for testing, and all remaining autosomes plus chrX for training.

The third and fourth datasets, referred to as the Zhang and Rao datasets, respectively, were used to evaluate HiC4D-SPOT’s ability to detect biologically relevant chromatin anomalies in real-world contexts. The Zhang dataset captures chromatin dynamics during the differentiation of human embryonic stem cells (hESCs) into ventricular cardiomyocytes across six time points and is publicly available under GEO SuperSeries GSE186958 [40]. The Rao dataset comprises six time points from an auxin-induced cohesin degradation and recovery experiment in human cells, available under GEO SuperSeries GSE104334 [41]. Both datasets were processed using the same pipeline as the Reed dataset, including conversion to .cool format at 10 kb resolution, ICE normalization, and extraction of 50$\times $50 submatrices along the diagonal with a 25-bin stride. The Zhang dataset was used to evaluate HiC4D-SPOT’s ability to detect extensive chromatin remodeling events, such as HERV-H-associated TAD boundary weakening near the embryonic stem cell-related gene (ESRG) during differentiation. The Rao dataset was used to assess the model’s ability to capture rapid chromatin loop disruptions and their restoration following auxin-mediated cohesin depletion and auxin withdrawal.

#### Simulated Hi-C datasets

The spatiotemporal autoencoder architecture is evaluated under controlled conditions using synthetic Hi-C data generated by embedding structural perturbations into existing baseline Hi-C datasets. These synthetic structural perturbations are produced by adapting the procedures proposed by Lun and Smyth [42] and Forcato et al. [43], emphasizing spatial contexts such as TADs and loops while accounting for weak nonspecific ligation events inherent in Hi-C data.

Each simulated chromosome contact map is a $NxN$ matrix, where $N$ corresponds to the number of bins covering the genomic region defined by resolution where an entry $(x,y)$ pair in the matrix reflects the interaction frequency between bins $x$ and $y$. To avoid redundant operations on symmetric entries $(x,y)$ and $(y,x)$, only the lower triangular region of the matrix is considered (where $x>y$) to apply perturbations, with the results mirrored to the upper triangular region. The region for the perturbation for a structural context $i$ is defined by the start bin $s_{i}$ and end bin $e_{i}$, with width $w_{i}=e_{i}-s_{i}+1$.

To simulate different TAD dynamics, four major perturbation types, add, split, shift, and strength change, are considered following the principle of power-law decay to recapitulate the power-law relationship between distance and abundance observed in real genomic data [7]. A tolerance range of 10 bins is included around each TAD boundary to avoid overlapping perturbations. To introduce a *de novo* TAD, interaction values within selected $[s_{i}, e_{i}]$ region are increased according to the power-law decay where the mean interaction $\mu _{(x,y)}$ between the $(x,y)$ pair is modeled as the additive model of two components:


(1) \begin{align*} \mu_{t,(x,y)} &= k_{t}\,(\lvert x-y\rvert + p)^{c_{t}}, \textrm{if } x,y \in [s_{i}, e_{i}], \end{align*}

(2) \begin{align*} \mu_{n,(x,y)} &= \begin{cases} k_{n}\,(\lvert x-y\rvert + p)^{c_{n}}, & \textrm{if } x,y \in [s_{i}, e_{i}] \textrm{ and } U < p_{\mathrm{noise}},\\ 0, & \textrm{otherwise}, \end{cases} \end{align*}

where $k_{t}$ is the baseline constant, $p$ is a small prior to avoid division by zero, and $c_{t}$ is the power-law exponents controlling the rate of decay with respect to the distance between bins $x$ and $y$. The noise term $\mu _{2,(x,y)}$ has its own baseline $k_{n}$ and decay exponent $c_{n}$, but is only applied if the random draw $U$ (uniformly distributed in $[0,1]$) falls below the noise probability $p_{\mathrm{noise}}$. The noise signal is added or subtracted with equal probability to the first signal component and the updated interaction replaces the original signal only if it is larger, ensuring that *de novo* TAD formation leads to a net increase in contact frequency in the specified region:


(3) \begin{align*} \mu_{(x,y)} &= \mu_{t,(x,y)} \;\pm\; \mu_{n,(x,y)}, \end{align*}

(4) \begin{align*} N^{\prime}_{(x,y)} &= \begin{cases} \mu_{(x,y)}, & \textrm{if } \mu_{(x,y)}> N_{(x,y)},\\ N_{(x,y)}, & \textrm{otherwise}, \end{cases} \end{align*}

where $N_{(x,y)}$ is the original interaction frequency at $(x,y)$, and $N^{\prime}_{(x,y)}$ is the updated interaction frequency after the perturbation.

A TAD split event partitions an existing TAD into multiple subdomains, reducing interactions between $[s_{i}, e_{i}]$. A weaker baseline $k_{t^{\prime}}$ replaces $k_{t}$ to reflect diminished contact frequencies in the newly separated region, and the noise term is always subtracted. The resulting interaction $\mu _{(x,y)}$ is used to update $N_{(x,y)}$ only when it is smaller, mirroring the loss of contact strength:


(5) \begin{align*} \mu_{t^{\prime},(x,y)} &= k_{t^{\prime}}\,(\lvert x-y\rvert + p)^{c_{t}}, \textrm{if } x,y \in [s_{i}, e_{i}], \end{align*}

(6) \begin{align*} \mu_{n,(x,y)} &= \begin{cases} k_{n}\,(\lvert x-y\rvert + p)^{c_{n}}, & \textrm{if } x,y \in [s_{i}, e_{i}] \textrm{ and } U < p_{\mathrm{noise}},\\ 0, & \textrm{otherwise}, \end{cases} \end{align*}

(7) \begin{align*} \mu_{(x,y)} &= \mu_{t^{\prime},(x,y)} \;-\; \mu_{n,(x,y)}, \end{align*}

(8) \begin{align*} N^{\prime}_{(x,y)} &= \begin{cases} \mu_{(x,y)}, & \textrm{if } \mu_{(x,y)} < N_{(x,y)},\\ N_{(x,y)}, & \textrm{otherwise}, \end{cases} \end{align*}

where $k_{t^{\prime}}$ is smaller than $k_{t}$ to emphasize reduced interaction strength in the weak region undergoing TAD splitting. The final value is only retained if $\mu _{(x,y)}$ is lower than the baseline $N_{(x,y)}$, ensuring that TAD splitting corresponds to a net decrease in local contact intensity.

A shift operation moves an entire TAD domain by a fixed offset to either side or a specific location, leaving behind a region of weaker interactions. The vacated region is modeled akin to the splitting procedure, employing the reduced baseline $k_{t^{\prime}}$ and subtractive noise term. The shifted TAD region is then superimposed onto its new location. This approach simulates TAD repositioning along the chromosome while preserving weak nonspecific ligation events in the original domain.

A strength change operation modifies the average contact intensity of an existing TAD without altering its location or structure. A global multiplier is applied to the TAD’s baseline (such as scaling $k_{t}$ by a factor of $0.5$ or $1.5$) to represent weakened or strengthened interactions, followed by the addition or subtraction of random noise. Similar to the add or split cases, the final interaction values are updated only when the new value is lower or higher, respectively, to reflect the intended direction of change (reduction or enhancement).

Similar to TAD perturbations, chromatin loops undergo strength-change operations. For the strength increase, a representative loop signature is generated by averaging the interaction patterns of the top five high-confidence loops located near the region of interest. This averaged loop matrix is then used to replace the interaction values in the target region when the new value exceeds the original, reflecting the intended increase in loop strength. An upper bound is imposed on the modified contact values to ensure numerical stability and biological plausibility. For the strength decrease, a rectangular window is selected around the loop of interest, expanded by two bins in each direction to capture its immediate surroundings. Each contact frequency within this window is then recalculated as the mean of four corresponding positions located above, below, left, and right of the loop region. This averaging ensures that any elevated interaction signals merge with local baseline values, reducing the loop’s intensity while maintaining symmetry by mirroring the updated entries. As a result, the contact map reflects a weaker loop signal that closely matches the surrounding genomic interaction landscape.

In the experiment, the TAD baseline ($k_{t}$) and power-law exponent ($c_{t}$) are set to 400 and −0.8, respectively, while the weaker baseline ($k_{t}$) is set to 200 with the same exponent. The offset ($p$) is fixed at 1, and the random noise parameters ($k_{n}$, $c_{n}$) are set to (50, −0.7), incorporated at a probability ($p_{\mathrm{noise}} $) of 0.5.

### Spatiotemporal autoencoder

The HiC4D-SPOT architecture, as illustrated in Fig. 1, implements a Convolution Long Short-Term Memory (ConvLSTM)-based autoencoder to extract compact representations of spatiotemporal Hi-C data by leveraging the synergistic interplay between convolutional and recurrent operations, thereby capturing both local spatial dependencies within each matrix and evolving temporal correlations across consecutive frames, making it well-suited for detecting anomalous contact patterns that deviate from expected chromatin interaction dynamics.

As depicted in Fig. 1, the HiC4D-SPOT framework comprises a spatial encoder, a temporal encoder–decoder using ConvLSTM, and a spatial decoder, which expects an input sequence $\mathbf{X} = \{\mathbf{X}_{1}, \mathbf{X}_{2}, \ldots , \mathbf{X}_{T}\}$ and outputs a reconstructed sequence $\widehat{\mathbf{X}} = \{\widehat{\mathbf{X}}_{1}, \widehat{\mathbf{X}}_{2}, \ldots , \widehat{\mathbf{X}}_{T}\}$, where each sequence has dimensions $T \times H \times W \times C$, with $T$ representing the number of time points, $H$ and $W$ denoting the spatial dimensions, and $C$ indicating the number of channels.

The spatial encoder block consists of multiple convolutional layers $l$ with stride and pooling, reducing spatial resolution while extracting hierarchical features on each frame:


(9) \begin{align*}& \mathbf{E}^{(l)} = f\!\Bigl(\mathrm{Conv}\bigl(\mathbf{E}^{(l-1)}; \mathbf{W}^{(l)}\bigr)\Bigr),\end{align*}

where $\mathbf{E}^{(0)} = \mathbf{X}$ is the original input sequence reshaped for convolution, $\mathbf{W}^{(l)}$ are the learnable filters, and $f(\cdot )$ is a nonlinear activation. The feature map from the last encoder layer is then passed to the temporal encoder–decoder block for spatiotemporal modeling.

The temporal encoder–decoder block, based on ConvLSTM cells, consists of a stack of ConvLSTM layers, where each ConvLSTM cell receives the current features $\mathbf{x}_{t}$, the previous hidden state $\mathbf{h}_{t-1}$, and the previous cell state $\mathbf{c}_{t-1}$. Its gating mechanism is described as follows:


(10) \begin{align*} & \mathbf{i}_{t} = \sigma\Bigl(\mathrm{Conv}(\mathbf{x}_{t}, \mathbf{W}_{x i}) + \mathrm{Conv}(\mathbf{h}_{t-1}, \mathbf{W}_{h i}) + \mathbf{b}_{i} \Bigr), \end{align*}

(11) \begin{align*} & \mathbf{f}_{t} = \sigma\Bigl(\mathrm{Conv}(\mathbf{x}_{t}, \mathbf{W}_{x f}) + \mathrm{Conv}(\mathbf{h}_{t-1}, \mathbf{W}_{h f}) + \mathbf{b}_{f} \Bigr), \end{align*}

(12) \begin{align*} & \tilde{\mathbf{c}}_{t} = \tanh\Bigl(\mathrm{Conv}(\mathbf{x}_{t}, \mathbf{W}_{x c}) + \mathrm{Conv}(\mathbf{h}_{t-1}, \mathbf{W}_{h c}) + \mathbf{b}_{c} \Bigr), \end{align*}

(13) \begin{align*} & \mathbf{o}_{t} = \sigma\Bigl(\mathrm{Conv}(\mathbf{x}_{t}, \mathbf{W}_{x o}) + \mathrm{Conv}(\mathbf{h}_{t-1}, \mathbf{W}_{h o}) + \mathbf{b}_{o} \Bigr), \end{align*}

where $\mathbf{i}_{t}, \mathbf{f}_{t},\ and\ \mathbf{o}_{t}$ are the input, forget, and output gates, $\tilde{\mathbf{c}}_{t}$ is the candidate cell state, $\sigma (\cdot )$ is the sigmoid function, and $\mathrm{Conv}(\cdot ,\cdot )$ denotes a 2D convolution. The cell states and hidden states are updated as


(14) \begin{align*} & \mathbf{c}_{t} = \mathbf{f}_{t} \odot \mathbf{c}_{t-1} \;+\; \mathbf{i}_{t} \odot \tilde{\mathbf{c}}_{t}, \end{align*}

(15) \begin{align*} & \mathbf{h}_{t} = \mathbf{o}_{t} \odot \tanh(\mathbf{c}_{t}), \end{align*}

where $\odot $ is element-wise multiplication.

The output from the final ConvLSTM layer is passed to the spatial decoder, which performs transposed convolutions to recover the original spatial resolution $H \times W$, where each decoder layer applies


(16) \begin{align*}& \mathbf{D}^{(m)} = f\!\Bigl(\mathrm{DeConv}\bigl(\mathbf{D}^{(m-1)}; \mathbf{W}^{(m)}\bigr)\Bigr),\end{align*}

where $\mathbf{D}^{(0)}$ is the ConvLSTM output, $\mathrm{DeConv}$ is a transposed convolution, and $f(\cdot )$ is a nonlinear function. After the last decoder layer, the reconstructed sequence $\widehat{\mathbf{X}}$ matches the input shape $T \times H \times W \times C$.

The AI model is trained to minimize the reconstruction error between the input sequence $\mathbf{X}_{t}$ and output sequence $\widehat{\mathbf{X}}_{t}$, with high error values indicating anomalous Hi-C contact patterns.


(17) \begin{align*}& \mathcal{L}_{\mathrm{reconstruction}} = \frac{1}{T\,H\,W\,C} \sum_{t=1}^{T} \Bigl\| \mathbf{X}_{t} - \widehat{\mathbf{X}}_{t} \Bigr\|_{2}^{2}.\end{align*}

### Implementation details

The HiC4D-SPOT model was implemented using PyTorch [44] and trained on a ConvLSTM-based autoencoder framework. The spatial encoder consists of three convolutional layers with kernel sizes of $ (7, 5, 3) $, strides of $ (2,2,1) $, and padding values of $ (3,2,1) $, followed by batch normalization and ReLU activation after each convolution operation. The temporal encoder–decoder employs a stack of five ConvLSTM layers, each with a kernel size of $ 3 $, hidden state dimensions of $ [64, 64, 32, 64, 64] $, preserving the local spatial structure while allowing effective temporal modeling across time points. Analogous to the spatial encoder, the spatial decoder is symmetric to the encoder, comprising three transposed convolutional layers with kernel sizes $ (3, 5, 7) $, strides $ (1,2,2) $, and padding $ (1,2,3) $, with batch normalization and ReLU applied after each layer, except for the final output layer, which uses a sigmoid activation function. A dropout of 0.3 is implemented inside each layer of every block to prevent overfitting.

The model was trained using the AdamW optimizer [45] with an initial learning rate and weight decay of 0.0001, and a batch size of 32. Training was conducted for a maximum of 100 epochs with early stopping based on validation loss, using a patience of 25 epochs. The training procedure was parallelized on a 40GB NVIDIA A100 GPU, significantly reducing computation time, with each epoch completing in $\sim $2 min compared with 2 h per epoch on a CPU. Inference speed was also optimized, reducing the computation time from 6.83 s per instance on a CPU to 0.35 s per instance on a GPU.

## Results

### Reconstruction performance

A high-fidelity reconstruction is essential for modeling normal chromatin interactions, allowing deviations from expected patterns to serve as an indicator of structural irregularities. To evaluate the reconstruction capability of HiC4D-SPOT, we assessed its ability to accurately recover Hi-C contact matrices across multiple developmental time points. In the experiment, the test split of time-series Hi-C data from the Du and Reed datasets—representing chromatin dynamics suitable for assessing overall reconstruction fidelity—was fed into the AI model, which generated reconstructed Hi-C matrices for each time point. The Zhang and Rao datasets were not included in this evaluation, as they contain localized, biologically meaningful chromatin anomalies that are the focus of downstream detection tasks and were used specifically to assess the model’s anomaly detection capability.

**Figure 2:**
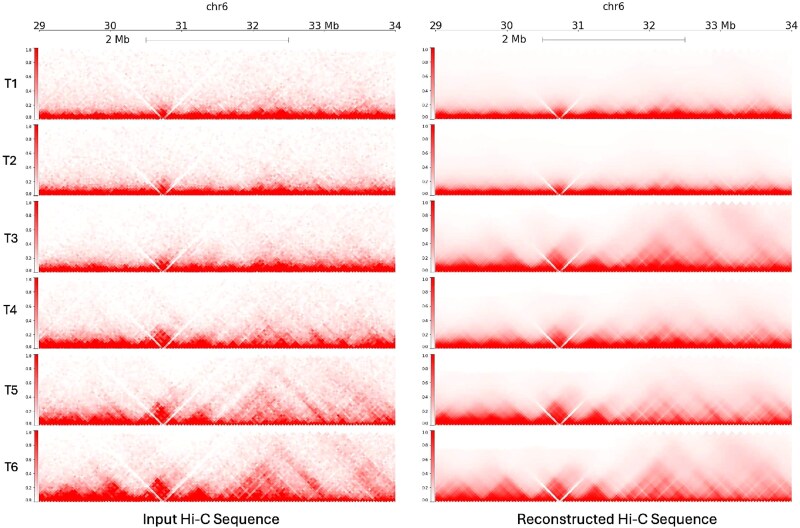
Visualization of HiC4D-SPOT’s performance on its ability to reconstruct spatiotemporal Hi-C data on the Du dataset for chromosome 6 across six developmental stages (T1–T6).

Figure 2 presents a qualitative comparison between the input and reconstructed Hi-C matrices on the Du dataset, illustrating the model’s ability to preserve key structural features. The reconstructed matrices exhibit a strong resemblance to the input, effectively maintaining chromatin interaction patterns, domain boundaries, and contact intensity gradients, thereby demonstrating the AI’s capacity to capture complex high-dimensional spatiotemporal dependencies inherent in chromatin architecture.

To quantitatively assess reconstruction performance, we computed multiple evaluation metrics, including mean squared error (MSE), L1 loss, structural similarity index (SSI), Pearson correlation coefficient (PCC), Spearman correlation coefficient (SCC), and other relevant metrics across different time points on both the Du and Reed datasets. The results, detailed in Table 1, indicate that the model consistently achieves high SSI and PCC values across all time points, reinforcing its ability to retain intricate spatial structures within Hi-C matrices. The low MSE and L1 loss values further substantiate the model’s precision in reconstructing chromatin interaction patterns with minimal distortion, ensuring the preservation of biologically relevant contact relationships. Notably, the model maintains an average SSI exceeding 0.99 and PCC above 0.98 across both datasets, underscoring its robustness in reconstructing Hi-C matrices while maintaining structural coherence across diverse genomic contexts.

**Table 1:** Reconstruction performance of HiC4D-SPOT on the Du and Reed datasets across different time points. The table reports reconstruction quality metrics, including MSE, L1 Loss, SSI, PCC, SCC, peak signal-to-noise ratio (PSNR), normalized root mean square error (NRMSE), Kullback–Leibler (KL) divergence, Jensen–Shannon (JS) divergence, and cosine similarity. The average performance across all time points is also reported for each dataset. Higher SSI, PCC, SCC, PSNR, and cosine similarity indicate better reconstruction fidelity, while lower MSE, L1 Loss, NRMSE, KL, and JS divergence signify improved accuracy.

Dataset	Time point	MSE	L1 Loss	SSI	PCC	SCC	PSNR	NRMSE	KL divergence	JS divergence	Cosine similarity
Du	1	$\sim $ 0.0	0.001	0.991	0.990	0.980	42.812	0.046	0.041	0.011	0.990
	2	$\sim $ 0.0	0.001	0.991	0.988	0.970	42.136	0.050	0.059	0.014	0.988
	3	$\sim $ 0.0	0.001	0.991	0.986	0.976	41.132	0.056	0.106	0.014	0.986
	4	$\sim $ 0.0	0.001	0.991	0.982	0.967	40.006	0.064	0.170	0.015	0.982
	5	$\sim $ 0.0	0.001	0.990	0.979	0.965	39.970	0.064	0.236	0.017	0.980
	6	$\sim $ 0.0	0.001	0.990	0.971	0.963	37.667	0.084	0.247	0.019	0.971
	Average	$\sim $ 0.0	0.001	0.991	0.983	0.970	40.621	0.061	0.143	0.015	0.983
Reed	1	$\sim $ 0.0	$\sim $ 0.0	0.997	0.994	0.960	51.126	0.018	0.027	0.007	0.994
	2	$\sim $ 0.0	$\sim $ 0.0	0.997	0.992	0.958	50.418	0.019	0.033	0.008	0.993
	3	$\sim $ 0.0	$\sim $ 0.0	0.997	0.994	0.960	50.931	0.018	0.032	0.007	0.994
	4	$\sim $ 0.0	$\sim $ 0.0	0.997	0.993	0.956	50.663	0.019	0.057	0.008	0.994
	5	$\sim $ 0.0	$\sim $ 0.0	0.997	0.993	0.952	50.458	0.020	0.079	0.009	0.993
	6	$\sim $ 0.0	$\sim $ 0.0	0.997	0.993	0.951	50.405	0.019	0.093	0.009	0.993
	7	$\sim $ 0.0	$\sim $ 0.0	0.996	0.993	0.950	50.282	0.020	0.103	0.009	0.993
	8	$\sim $ 0.0	$\sim $ 0.0	0.996	0.989	0.944	48.831	0.020	0.119	0.013	0.989
	Average	$\sim $ 0.0	$\sim $ 0.0	0.997	0.993	0.954	50.389	0.019	0.068	0.009	0.993

### Detection of spatiotemporal deviations in time-swap experiment

**Figure 3:**
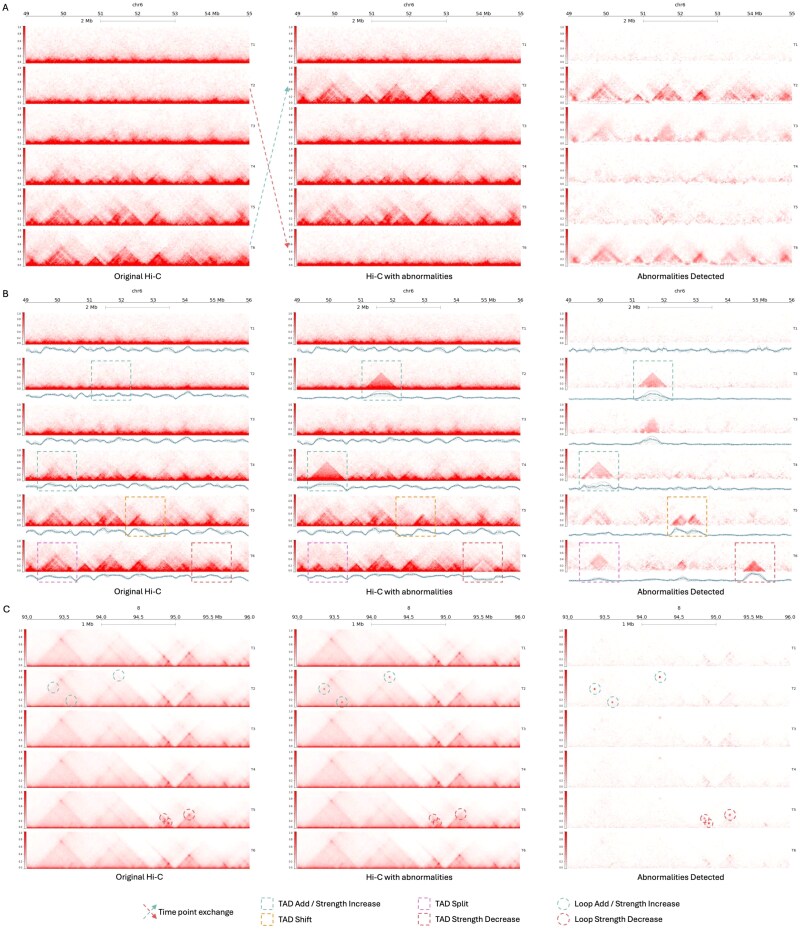
Visualization of HiC4D-SPOT’s performance, depicting the original Hi-C, Hi-C with abnormalities, and abnormalities detected on: (A) Du dataset across six developmental stages (T1–T6) for chromosome 6, with T2 and T6 swapped in the input to assess the model’s ability to capture significant spatiotemporal deviations; (B) Du dataset across six developmental stages (T1–T6) for chromosome 6, with simulated TAD add, strength change, split, and shift perturbations introduced to evaluate the model’s ability to detect SVs in spatiotemporal Hi-C data; (C) Reed dataset across six stages for chromosome 8, with simulated loop strength change to assess the model’s ability to detect abnormal chromatin loop interactions.

HiC4D-SPOT’s ability to detect temporal inconsistencies was assessed using a time-swap experiment on the Du dataset for chromosome 6 in which time points T2 and T6 were deliberately interchanged within the original Hi-C sequence, as shown in Fig. 3A, serving as a controlled test to evaluate whether the model could distinguish deviations in temporal chromatin interaction patterns. The time-swapped time-series Hi-C data were input into the model trained on the training split of the Du dataset to get the expected reconstruction of the original Hi-C matrices. The reconstructed Hi-C matrices were then compared against the input time-swapped Hi-C data, with the discrepancies between the two serving as the abnormalities detected by our tool. Figure 3A illustrates the original Hi-C, Hi-C with abnormalities, and abnormalities detected across six developmental stages from the time-swap experiment, where anomalous regions corresponding to the swapped time points are visibly detected and distinct from their neighboring unperturbed time points, highlighting the model’s ability to capture spatiotemporal aberrations in the expected chromatin interaction trajectory.

**Figure 4:**
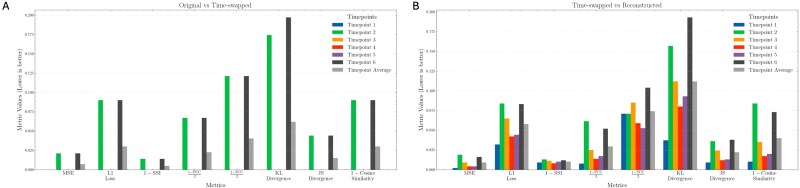
Grouped bar plots illustrating the performance of HiC4D-SPOT across different metrics for each time point in the Du dataset with T1–T6 time-swapped Hi-C on chromosome 6: (A) original versus time-swapped and (B) time-swapped versus reconstructed.

This is further supported by the quantitative evaluation metrics presented in Fig. 4A, where the evaluation metrics comparing the original Hi-C and time-swapped Hi-C showcase the evidence of perturbation on the original Hi-C, providing the expected anomaly score for the deviated time points. Taking Fig. 4A as a reference, Fig. 4B quantifies the deviations observed between time-swapped Hi-C and the reconstructed expected original Hi-C for each time point, revealing significant divergence from the average baseline values, with anomaly scores for T2 and T6 consistently elevated relative to other time points and closely align to the expected values from Fig. 4A. This increase in anomaly score from the baseline score indicates that the AI model successfully identified inconsistencies arising from temporal displacement, demonstrating its sensitivity to alterations in the expected chromatin interaction trajectory.

### Anomaly detection of simulated SVs

In addition to the assessment of the model on reconstruction and temporal consistency, we further assessed its ability to detect SVs in spatiotemporal Hi-C data under simulated conditions, where the AI model was tasked with identifying anomalous chromatin structural changes introduced into the original Hi-C matrices. The simulated structural perturbations emulate biologically plausible changes in chromatin organization—such as TAD splitting, TAD shifting, TAD strength modulation, and loop strength alterations—that have been observed in various biological contexts and interventional experiments, including cancer and disease progression, and genome engineering using CRISPR-Cas9 [41, 46–51]. These controlled perturbations serve as quantifiable benchmarks to assess the model’s sensitivity to localized structural anomalies in 3D genome architecture.

In this experiment, the Hi-C matrices subjected to simulated structural perturbations were input into the model to get the reconstruction of the expected original Hi-C matrices, where the difference between the input and reconstructed spatiotemporal Hi-C matrices provided a measure to detect anomalous regions.

Firstly, the model, trained on the Du dataset, is evaluated for the detection of anomalous TAD under the controlled environment by simulating anomalous TAD dynamics across multiple time points on chromosome 6 of the Du dataset, such as additions, strength changes, shifts, and splits, as illustrated in Fig. 3B. The abnormalities detected section in Fig. 3B exhibit precise identification of the introduced TAD perturbations, with the model successfully flagging regions of altered chromatin structure as anomalous.

To quantitatively validate the detection of these perturbations in Fig. 3B, TAD-calling tools were employed to analyze the Hi-C matrices. The TAD-separation score, computed using hicFindTADs [52], provided a z-score-based measure of TAD boundary strength, visualized beneath each Hi-C map in Fig. 3B. The separation score confirms the presence of SVs, with distinct shifts in separation scores observed in the loci of detected abnormalities, where perturbed regions exhibit altered boundary patterns indicative of abnormal chromatin reorganization.

Additionally, to further validate the model’s capability to detect anomalous SVs, OnTAD [53] was used to detect the presence of perturbations in the abnormalities detected regions, yielding high-confidence identification of simulated TAD regions. The TAD strength increases at time points T2 and T4 were detected with confidence scores of 4.561 and 3.701, respectively, the TAD shift at T5 had a confidence score of 3.394, and the TAD split and TAD strength decrease at T6 were detected with scores of 0.856 and 3.894, respectively. The average OnTAD confidence score for simulated TADs ranks in the 99th percentile relative to TADs detected in the original Hi-C, indicating that the detected perturbations represent structurally significant variations. A Mann–Whitney U test confirms that the confidence scores of the simulated TADs are significantly higher than the typical TAD scores, with a $P$-value of 0.00019, demonstrating that the detected SVs are statistically distinct from normal TAD structures. These findings reinforce the model’s ability to capture abnormal structural changes, offering valuable insights into the detection of dynamic 3D genome organization anomalies.

Secondly, the model, trained on the Reed dataset, is evaluated for the detection of anomalous loops under the controlled environment by simulating anomalous loop dynamics across multiple time points on chromosome 8 of the Reed dataset, such as loop strength changes, as illustrated in Fig. 3C. The abnormalities detected exhibit precise identification of the introduced loop perturbations, with the model successfully flagging regions of altered chromatin loop interactions as anomalous. This is supported by the implementation of Mustache [54], a loop calling tool, to extract statistically significant (P_t_ = 0.01) loops from the sequence of detected anomalous regions, where all the simulated loops were identified with low false discovery rates (FDR $< 1.44 \times 10^{-6}$), confirming their statistical significance. This validates the model’s ability to detect abnormal chromatin loop interactions with high statistical confidence, underscoring its potential for investigating chromatin loop dynamics and their roles in cellular functions.

### Detection of dynamic chromatin structural anomalies in real-world data

To evaluate HiC4D-SPOT’s capability in detecting biologically meaningful structural anomalies in real-world data, we applied it to the Zhang dataset, which captures the transition of hESCs into ventricular cardiomyocytes, a process accompanied by extensive chromatin reorganization [40]. The differentially expressed genes are expected to be enriched near dynamic TAD boundaries, particularly in regions associated with the human endogenous retrovirus subfamily H (HERV-H), which plays a key role in maintaining chromatin structure and pluripotency [56]. Among these genes, the ESRG is a long noncoding RNA whose transcription is tightly regulated by HERV-H activity and is essential for maintaining stem cell identity. HERV-H elements establish strong chromatin insulation in hESCs (Day 0), reinforcing the boundaries of regulatory regions and preventing unwanted interactions between adjacent loci. However, their boundary strength weakens during differentiation, leading to chromatin reorganization that facilitates lineage-specific transcriptional programs, including the regulation of ESRG expression. This provides an ideal biological framework to assess whether the model can capture these highly dynamic and functionally relevant chromatin transitions.

**Figure 5:**
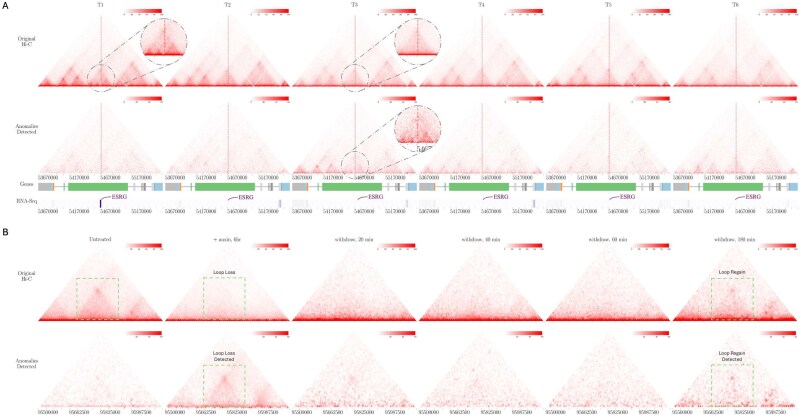
Visualization of HiC4D-SPOT’s performance on real-world data: (A) the six time point human cardiomyocyte differentiation time course from hESC to ventricular cardiomyocytes, highlighting dynamic chromatin anomalies near HERV-H associated loci, particularly at the ESRG gene, an HERV-H-associated lncRNA linked to pluripotency regulation; (B) cohesin depletion dataset, capturing loop domain disruptions following auxin-mediated cohesin degradation and restoration, with green boxes highlighting anomalous regions; heatmaps were generated using a modified version of the visualization script from [55].

The differentiation dataset was input into the model, trained on the Reed dataset, to get the expected chromatin interaction dynamics across six time points. As shown in Fig. 5A, at time point T1, ESRG is highly expressed, and its associated TAD boundary is well-defined, exhibiting multiple small TADs, indicative of strong insulation and localized chromatin compartmentalization. However, by time point T3, boundary strength at the ESRG locus weakens substantially, leading to a loss of insulation and the formation of a single large TAD. This boundary dissolution aligns with previously reported HERV-H-associated weakening, indicating a chromatin reorganization event. The model effectively captures this loss of insulation, as depicted in Fig. 5A, suggesting that chromatin reorganization at ESRG-associated regions is functionally linked to HERV-H activity. This transition reflects a key shift in stem cell identity, where boundary weakening facilitates the activation of lineage-specific genes, contributing to the observed chromatin remodeling during differentiation.

Additionally, we evaluated the performance of HiC4D-SPOT, trained on the Reed dataset, on real-life interventional experiment on hg19 genome, the Rao dataset, where auxin-mediated degradation of cohesin leads to the rapid loss and subsequent recovery of chromatin loop domains and the cohesin depletion results in near-complete loop disappearance within 60 min, with reformation upon auxin withdrawal [41]. In such temporal experiments, it is essential to identify the regions undergoing significant irregular structural changes, as these can be indicative of crucial cellular dysfunctions. As depicted in Fig. 5B, the AI model precisely identifies regions undergoing these anomalous transitions, effectively capturing the loss and re-establishment of loop domains over time with high precision, distinguishing anomalous structural changes from normal chromatin dynamics. By pinpointing loci with transient loop loss and reformation, the HiC4D-SPOT provides a powerful framework for analyzing genome architecture under perturbative conditions, demonstrating its utility in studying chromatin remodeling linked to gene regulation, and differentiation.

## Discussion and conclusions

In this paper, we present HiC4D-SPOT, a computational tool for unsupervised detection of anomalous chromatin interaction patterns in spatiotemporal Hi-C data. By leveraging ConvLSTM-based autoencoder, our method demonstrates significant performance across different conditions, effectively identifying anomalous structural deviations in chromatin organization with high accuracy and sensitivity.

HiC4D-SPOT processes Hi-C data temporally, capturing both spatial chromatin organization and its dynamic transition across time points, enabling the detection of anomalous chromatin interactions that deviate from expected genomic dynamics. The tool accurately reconstructs the expected chromatin dynamics across diverse datasets, with low error and strong similarity metrics, demonstrating high reconstruction fidelity and the ability to capture chromatin organization with spatial and temporal coherence. This fidelity in modeling expected chromatin dynamics underscores its capacity to discern structural anomalies, as deviations from the learned spatiotemporal patterns manifest as detectable perturbations, distinguishing abnormal SVs from normal chromatin dynamics.

The tool demonstrates significant accuracy in detecting spatiotemporal anomalies, as evidenced by its ability to identify anomalies in various simulated and real-world datasets. HiC4D-SPOT successfully captured temporal inconsistencies arising from time-swap experiments with high intensity, accurately detecting swapped time points and flagging them as anomalous. Similarly, HiC4D-SPOT effectively identified simulated TAD and loop perturbations, demonstrating its sensitivity to SVs in chromatin organization. Its performance on real biological datasets further validates its utility in detecting dynamic chromatin anomalies linked to differentiation and perturbation, with high anomaly scores near HERV-H associated loci in the cardiomyocyte differentiation dataset and accurate tracking of loop domain changes in the cohesin depletion dataset.

These results establish HiC4D-SPOT as a powerful framework for analyzing 3D chromatin dynamics, capable of detecting both biologically driven and experimentally induced structural anomalies. While HiC4D-SPOT effectively identifies spatiotemporal chromatin anomalies, the model functions as a black-box system, making it challenging to interpret the underlying genomic features driving detected anomalies. Therefore, further research is needed to elucidate the gap between computational inferences and biological interpretations, enabling the model to provide actionable insights into the functional significance of detected chromatin anomalies.

**Key Points**

- We present HiC4D-SPOT, the first deep learning framework designed to detect structural anomalies in spatiotemporal Hi-C data using a ConvLSTM-based autoencoder, addressing a critical gap in current chromatin analysis tools.
- The model achieves high reconstruction fidelity across datasets (PCC and SCC > 0.9), showcasing accurate modeling of normal chromatin interaction dynamics.
- HiC4D-SPOT accurately detects chromatin anomalies in both simulated and real-world datasets, capturing temporal inconsistencies, structural perturbations in TADs and loops, and biologically validated chromatin remodeling events.
- Statistical validations confirm the structural significance of detected anomalies, establishing HiC4D-SPOT as an efficient framework for detecting and analyzing dynamic, and functionally relevant chromatin anomalies.

## Data Availability

The source code of HiC4D-SPOT along with scripts to generate the plots are available on the GitHub repository: https://github.com/zwang-bioinformatics/HiC4D-SPOT.
